# Distance dependent charge separation and recombination in semiconductor/molecular catalyst systems for water splitting†
†Electronic supplementary information (ESI) available: Experimental details, DFT calculations and additional transient absorption measurements. See DOI: 10.1039/c4cc05143b



**DOI:** 10.1039/c4cc05143b

**Published:** 2014-09-10

**Authors:** Anna Reynal, Janina Willkomm, Nicoleta M. Muresan, Fezile Lakadamyali, Miquel Planells, Erwin Reisner, James R. Durrant

**Affiliations:** a Department of Chemistry , Imperial College London , Exhibition Road , London SW7 2AZ , UK . Email: a.reynal@imperial.ac.uk ; Email: j.durrant@imperial.ac.uk; b Christian Doppler Laboratory for Sustainable SynGas , Department of Chemistry , University of Cambridge , Cambridge CB2 1EW , UK . Email: reisner@ch.cam.ac.uk

## Abstract

The photoinduced reduction of three Co electrocatalysts immobilised on TiO_2_ is 10^4^ times faster than the reverse charge recombination. Both processes show an exponential dependence on the distance between the semiconductor and the catalytic core.

## Abstract 2

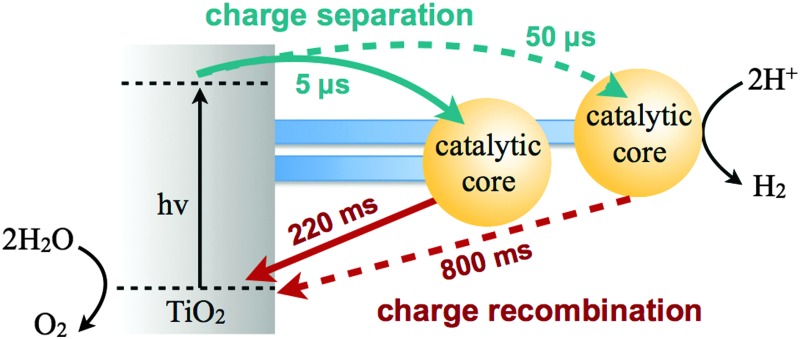
The molecular structure of the catalyst strongly influences the kinetics of charge separation and recombination.

The immobilisation of molecular catalysts on semiconductors for solar fuel production is an attractive strategy to exploit electrocatalysts in a heterogeneous photocatalytic environment. Efficient H_2_ production in such hybrid systems requires effective electronic coupling between the light harvesting unit and the electrocatalyst. Current examples based on non-precious metal complexes include Co, Ni and Fe electrocatalysts attached to narrow band-gap or dye-loaded wide band-gap semiconductors that allow for visible light absorption.^1^ In order to reduce protons to H_2_ through a mononuclear heterolytic route, the semiconductors have to transfer two electrons to one molecular catalyst. Previously, we reported that recombination of the reduced catalyst with valence band holes in the semiconductor limits the efficiency in these photocatalytic systems.^2^ Thus, achieving essentially uni-directional (vectorial) electron transfer from the semiconductor to the catalyst is crucial for enhancing long-lived charge separation and allowing the slow catalytic reactions to take place before electron–hole recombination.

Understanding and controlling the influence of the molecular structure on interfacial electron transfer dynamics has been a key requirement to enhance the efficiency of dye sensitised solar cells (DSSCs).^3^ Analogously to DSSCs, one might expect that changes in the molecular structure of the catalyst in such hybrid systems for solar fuels will also affect the kinetics of charge separation and recombination^4^ (in reverse direction of charge separation compared to DSSCs, see Fig. 1). However, systematic studies addressing the effect of molecular structure of the catalyst on charge transfer dynamics are scarce. In this study, we compare the kinetics of charge separation and recombination when a semiconductor (TiO_2_) is functionalised with three related cobalt electrocatalysts, whose molecular structure varies the physical separation between the catalytic core and the semiconductor surface (Fig. 1). In this hybrid system, the semiconductor acts as light harvester and the H_2_ evolution is driven by the anchored molecular catalyst.

**Fig. 1:**
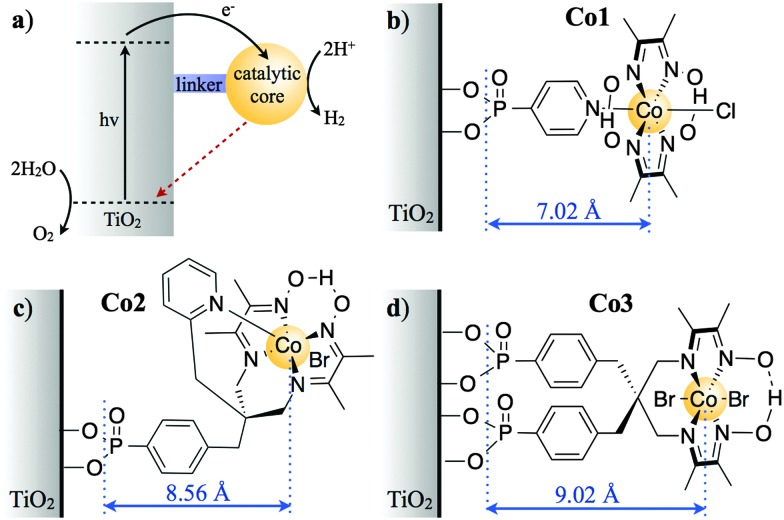
(a) Electron transfer processes in TiO_2_ functionalised with a molecular catalyst for H_2_ production after UV-light excitation. The solid black and dashed red arrows indicate charge separation and recombination, respectively. Molecular structures of the catalysts for H^+^ reduction are shown in (b) for **Co1**, (c) for **Co2** and (d) for **Co3** (charges omitted for clarity). The blue arrows indicate the distance between the anchoring groups and the catalyst metal centre (*r*, Å), as determined by energy minimised DFT calculations (Fig. S1, ESI†).‡
‡DFT calculations at B3LYP/6-311G(d) level of theory were performed to determine the spatial separation between the Co metal centre and phosphate groups anchoring these catalysts to the semiconductor at their most stable configuration/conformation (minimum energy).

The molecular catalysts and nanocrystalline anatase TiO_2_ films employed herein were synthesised as reported elsewere.^5^ Functionalisation of the TiO_2_ films with a monolayer of molecular catalyst (*ca.* 900 molecules of **Co1**, 1000 of **Co2** and 1050 of **Co3** per TiO_2_ particle, see ESI† for detailed calculations) was achieved by dipping the films into 10^–4^ M catalyst aqueous solutions for 12 h at rt in the dark. The kinetics of charge separation were studied by monitoring the photogenerated charge carriers (electrons and holes) in the nanostructured TiO_2_ films by transient absorption (TA) spectroscopy with a set-up described previously.^5*d*^ No signals directly from the Co catalysts were apparent over the spectral range studied. The signal corresponding to holes in TiO_2_ has a maximum at 460 nm, and electrons can be monitored at 900 nm.^6^

Electron transfer from the semiconductor to the molecular Co catalyst was first confirmed by TA measurements in the presence of a 0.1 M triethanolamine (TEOA) solution as hole scavenger. A lifetime of photoexcited TiO_2_ electrons of ∼1 s was observed on bare TiO_2_ films due to suppression of electron–hole recombination in the semiconductor. When TiO_2_ was functionalised with a Co electrocatalyst, the lifetime of photogenerated electrons in the semiconductor is reduced by ≥3 orders of magnitude, assigned to interfacial electron transfer to the molecular catalyst (Fig. 2). We note significant differences in the kinetics of this electron transfer upon the molecular catalyst employed. Thus, *t*_50%_ is ∼5 μs for **Co1**, 4 times slower for **Co2** (*t*_50%_ ∼ 20 μs) and 10 times slower for **Co3** (*t*_50%_ ∼ 50 μs) (Fig. S2, ESI† and further discussed below).

**Fig. 2:**
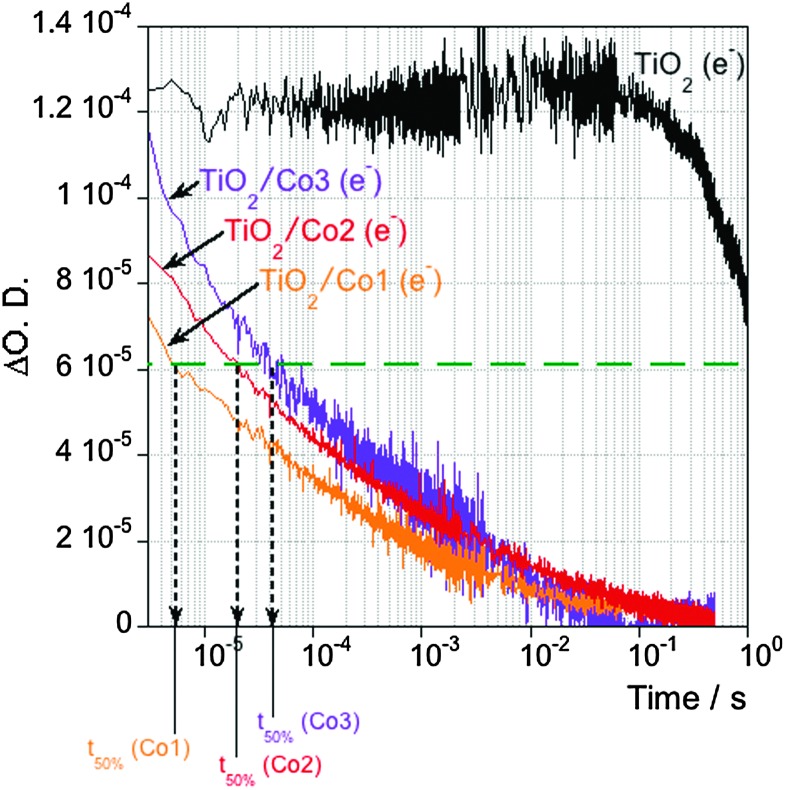
TA decays corresponding to photoexcited electrons in the TiO_2_ for bare films (black trace) and when functionalised with **Co1** (orange trace), **Co2** (red trace) and **Co3** (purple trace), measured in the presence of TEOA (0.1 M, buffered at pH 7) as hole scavenger.

The kinetics of charge recombination of electrons transferred to the catalytic centre with TiO_2_ valence band holes is only observed in the absence of chemical scavengers, so that the holes are not removed from the semiconductor. Conditions free of sacrificial agents are particularly relevant to coupling catalytic proton reduction to water oxidation, where the slow timescales of water oxidation will result in significant hole accumulation on the semiconductor. In an aqueous solution without hole scavenger, the decay of photogenerated electrons and holes in bare TiO_2_ presents identical kinetics on the micro- to milli-second timescales assigned to bimolecular recombination of these charge carriers through trapping–detrapping processes (Fig. S3, ESI†).^7^ Consistent with previous studies, band-gap excitation of the catalyst-loaded TiO_2_ showed smaller signal amplitudes and faster decays for photoexcited electrons compared to the bare metal oxides (Fig. 3).^2^ This behaviour is assigned to the transfer of electrons from the semiconductor to the molecular catalyst, whereas the holes remain in the valence band of the semiconductor. The decays of photoexcited holes are biphasic, with a fast phase (10^–6^–10^–4^ s) fitting to a power law equation and a slow component (10^–4^–1 s) fitting to a mono-exponential decay. This biphasic behaviour is clearest for **Co3**, where these two decay phases exhibit the largest difference in timescales, but is also resolved in fits to the decays for **Co1** and **Co2**, as shown by the dashed lines in Fig. 3. The fast, power law, hole decay phase exhibits similar kinetics to the decay of the electron signal at 900 nm, suggesting this phase should be assigned primarily to electron–hole recombination in the semiconductor, in competition with electron transfer to the molecular catalyst. The slow phase is assigned to recombination of long-lived holes in the valence band of the semiconductor with electrons transferred to the catalyst.§
§The relatively large amplitude of the fast hole decay phase for **Co1** and **Co2** suggests that this decay phase may also result in part from holes recombining with a sub-population of reduced catalysts unfavourably aligned relative to the TiO_2_ surface. The smaller amplitude of this phase for **Co3** is consistent with this catalyst employing two phosphonate linker units, ensuring alignment of all catalyst molecules normal to this surface. The timescale of this recombination reaction between the reduced catalyst and the holes in the semiconductor varies between catalysts, taking place in *t*_50%_ ∼ 220 ms for **Co1**, while being slower for **Co2** (*t*_50%_ ∼ 450 ms) and **Co3** (*t*_50%_ ∼ 800 ms) (determined from the time constant of the slow hole decay phase, see Fig. S4, ESI† for details of data fitting).

**Fig. 3:**
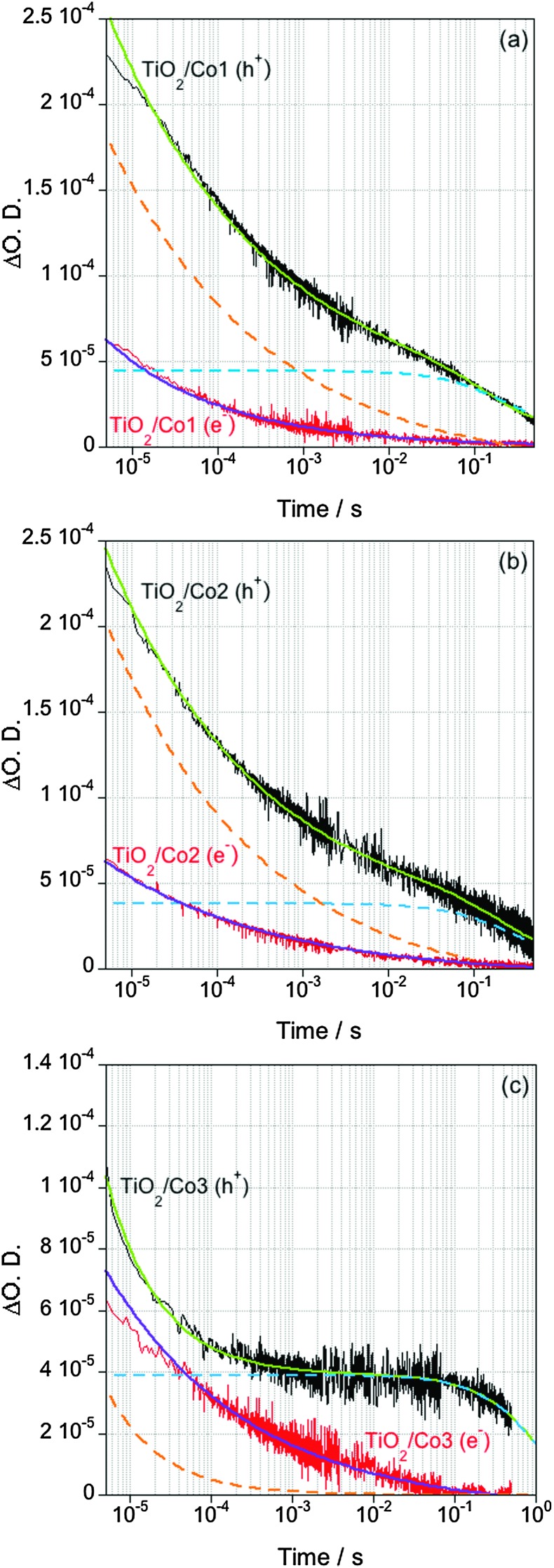
TA decays of TiO_2_ loaded with (a) **Co1**, (b) **Co2** and (c) **Co3**, measured in the absence of hole scavenger. The red traces correspond to electrons, while the black traces are assigned to holes. The orange and blue dashed lines represent the power law and exponential components of the biphasic holes decay, respectively.

The electron transfer between the semiconductor and the molecular catalyst is expected to occur through electron tunnelling, with a rate constant (*k*_ET_) depending exponentially upon the spatial separation between the semiconductor and the redox active orbitals of the molecular catalyst (*r*) – in this case, the metal centre – (*k*_ET_ ∝ e^–*βr*^, where *β* corresponds to the barrier height to electron tunnelling).^3a,8^ Fig. 4 shows the linear correlation between the log(1/*t*_50%_) and *r* for both charge separation from TiO_2_ to the catalyst (Fig. 2) and the recombination reaction between the reduced catalyst and the holes accumulated in the TiO_2_ valence band (slow hole decay phase, Fig. 3). Comparison of the >4 fold retardation of the electron transfer kinetics yields a value for the electron tunnelling exponent (*β*) of 1.12 Å^–1^ for the direct electron transfer and 0.65 Å^–1^ for the recombination reaction, lying within the calculated range for heterogeneous electron transfers through electron tunnelling across covalent bonds (∼0.5 to ∼1 Å^–1^).^8a,c^ Qualitatively similar behaviour was obtained using different kinetic analyses, with *β* ranging from 0.91–1.12 Å^–1^ for direct electron transfer and 0.51–0.65 Å^–1^ for the recombination reaction (Fig. S5 and Tables S1 and S2, ESI†). For the direct electron transfer, the value of *β* is similar to the distance dependencies observed previously for dye sensitised charge separation and recombination.^8*b*^ The origin of the somewhat smaller value of *β* for the recombination electron transfer reaction is unclear, but may be related to the energetics of the bridge group and/or some structural reorientation of the catalyst following electron transfer.^9^

**Fig. 4:**
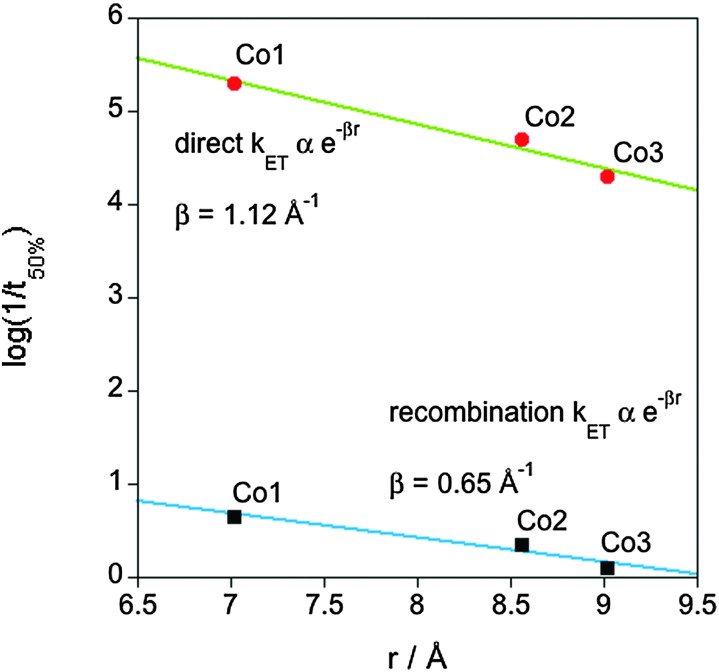
Plot of the logarithm of 1/*t*_50%_ of the electron transfer from TiO_2_ to the molecular catalyst (green trace) and the recombination between the reduced catalyst and the holes accumulated in the TiO_2_ (blue trace) as a function of catalyst core-anchoring groups distance.

It is apparent that the kinetics of photoinduced charge separation from the TiO_2_ conduction band to the catalytic centre is approximately 4 orders of magnitude faster than the reverse charge recombination with valence band holes. This rectifying behaviour is clearly beneficial for device function and is analogous to the rectifying behaviour observed at dye-sensitized interfaces.^8a,c^ The physical origin of this beneficial behaviour is not fully established. It may in part be associated with the recombination reaction lying in the Marcus inverted region (we estimate energetic driving energies for the forward and reverse electron transfer reactions of ∼0.6 and 2.7 eV respectively, in comparison the reorganisation energy for analogous systems is typically estimated as ∼1 eV^10^). It may also be associated with the relatively low density, and low mobilities, of valence band TiO_2_ holes. In either case, the relatively slow recombination dynamics is promising for future device development.

We note that the trends in electron transfer rate constants do not appear to correlate with differences in catalyst reduction potential. **Co3** exhibits a modestly more positive Co^III/II^ reduction potential than **Co1** and **Co2** (Table S3, ESI†). Assuming a reorganization energy of 1 eV,^10^ charge separation and recombination should exhibit normal and inverted dependencies, respectively, in contrast to our observation that **Co3** exhibits slower kinetics for both reactions. Rather for the catalyst series studied herein (where the differences in reduction potential are relatively small), the primary determinant of the rate constants appears to be the tunnelling distance. A detailed analysis of this point is beyond the scope of this paper.

The results herein demonstrate that semiconductor/catalyst interfaces can be effective at achieving charge separation in hybrid systems by physically separating the charge carriers, with the holes resting in the semiconductor and the electrons being transferred away from its surface. We note that molecular catalysts are not rigid structures and a distribution of lengths might coexist, thus, the calculated distances are considered a representation of the average distribution in our systems. The increase in lifetime is achieved in the absence of band bending within the semiconductor (not present herein as the TiO_2_ particle diameters are less than the space charge layer depth). The carrier lifetimes are increased as the spatial distance of the catalytic site from the semiconductor surface is increased. However, for the simple catalyst series studied herein, this increased lifetime comes at the expense of slower charge separation kinetics which, in the absence of hole scavengers, reduces the yield of charge separation. Strategies to mitigate this loss of yield could include appropriate design of linker energetics, or increased driving force for charge separation. Nevertheless, the reduction in electron–hole recombination achieved with **Co3** shows a remarkable potential for its use in hybrid systems for light-driven fuel synthesis reactions.

In summary, we have demonstrated the importance of catalyst molecular design to achieve long-lived charge separated states in hybrid molecular–semiconductor systems. The key parameter controlling the kinetics of charge separation and recombination is shown to be the physical separation between the semiconductor and the catalytic core. Thus, the introduction of linkers enhancing the distance between the semiconductor and the catalytic core allows for reduced electron–hole recombination by a factor of 4. This long-lived charge separation is likely to be crucial in performing the slow multi-electron catalytic reduction reaction of protons.

Financial support from the ERC (project Intersolar to J. D.) and the EPSRC (EP/H00338X/2 to E. R.) is gratefully acknowledged. A. R. thanks the European Commission Marie Curie CIG (PhotoCO2) and E. R. the Christian Doppler Research Association (Austrian Federal Ministry of Science, Research and Economy and National Foundation for Research, Technology and Development), and the OMV Group. We thank Shababa Selim and Ernest Pastor for experimental support.

## References

[cit1] Tran P. D., Wong L. H., Barber J., Loo J. S. C. (2012). Energy Environ. Sci..

[cit2] Reynal A., Lakadamyali F., Gross M. A., Reisner E., Durrant J. R. (2013). Energy Environ. Sci..

[cit3] Clifford J. N., Palomares E., Nazeeruddin M. K., Grätzel M., Nelson J., Li X., Long N. J., Durrant J. R. (2004). J. Am. Chem. Soc..

[cit4] Gao Y., Zhang L., Ding X., Sun L. (2014). Phys. Chem. Chem. Phys..

[cit5] (c) Willkomm J., Muresan N. M. and Reisner E., in preparation.

[cit6] Tang J., Durrant J. R., Klug D. R. (2008). J. Am. Chem. Soc..

[cit7] Nelson J. (1999). Phys. Rev. B: Condens. Matter Mater. Phys..

[cit8] (c) Miller R. J. D., McLendon G. L., Nozik A. J., Schmickler W. and Willig F., Surface electron transfer processes, VCH, New York, 1995.

[cit9] Winkler J. R., Gray H. B. (2014). J. Am. Chem. Soc..

[cit10] Solis B. H., Hammes-Schiffer S. (2011). Inorg. Chem..

