# Supplementary material for: Distance dependent charge separation and recombination in semiconductor/molecular catalyst systems for water splitting
Source: Chem Commun (Camb). 2014 Sep 10;50(84):12768–71. doi: 10.1039/c4cc05143b (PMC4183993; doi:10.1039/c4cc05143b)
Supplement: Supplementary file 1 [file CC-050-C4CC05143B-s001.pdf]

Electronic Supplementary Material (ESI) for ChemComm.
This journal is © The Royal Society of Chemistry 2014

Supporting information

# Distance dependent charge separation and recombination in semiconductor / molecular catalyst systems for water splitting

Anna Reynal,[a] Janina Willkomm,[b] Nicoleta M. Muresan,[b] Fezile Lakadamyali,[b] Miquel Planells,[a] Erwin Reisner,[b] and James. R. Durrant [a]

[a] Department of Chemistry, Imperial College London, Exhibition Road, London SW7 2AZ, UK.

[b] Christian Doppler Laboratory for Sustainable SynGas Chemistry, Department of Chemistry, University of Cambridge, Cambridge, CB2 1EW, UK.

**Contents** **Page**

Experimental details S1
Figures S1-S5 S2
Table S1-S3 S7
Additional references S7

## EXPERIMENTAL DETAILS

### Synthesis

Chemicals were purchased from commercial suppliers from the highest purity available and used without further purification.

$(Et_3NH)[Co^{III}Cl(dimethylglyoxime)_2(pyridyl\text{-}4\text{-}hydrophosphonate)$ (**Co1**), $[Co^{III}Br((DO)(DOH)(4\text{-}BnPO_3H_2)(2\text{-}CH_2py)\text{-}pn)]Br$ (**Co2**) and $[Co^{III}Br_2((DO)(DOH)(4\text{-}BnPO_3H_2)_2)\text{-}pn]$ (**Co3**), were prepared as described previously.[S1-S3]

Anatase $TiO_2$ films were deposited onto microscope or fluorine-doped tin oxide (FTO) coated glass by the Doctor blading technique from their respective colloidal pastes and calcined in a furnace at 450 °C for 30 min. The anatase $TiO_2$ paste was prepared as described in the literature.[S4] The films employed herein have an area of 1 cm x 1.5 cm and a thickness of 4 μm, determined by profilometry.

$TiO_2$ films loaded with a monolayer of catalyst were prepared by dipping the films into a $10^{-4}$ M catalyst aqueous solution for 12 h, at r.t. and in the dark to avoid degradation. The films were rinsed with distilled water to remove the excess of molecules not anchored onto $TiO_2$ prior to measurement.

### Transient absorption spectroscopy measurements

The microsecond-second transient absorption decays were measured using a Nd:YAG laser (Big Sky Laser Technologies Ultra CFR Nd:YAG laser system, 6 ns pulse width). The third harmonic of the laser, corresponding to 355 nm, at a frequency of 1 Hz, was used as excitation pulse. Typical excitation densities of 350 - 500 $\mu J/cm^2$ were used. A liquid light guide with a diameter of 0.5 cm was used to transmit the laser pulse to the sample. The probe light source was a tungsten lamp (Bentham IL1 tungsten lamp), and the probing wavelength was selected by using a monochromator (OBB-2001 dual grating, Photon Technology International) placed prior to the sample. Transient absorption data was collected with a Si photodiode (Hamamatsu S3071). The information was passed through an amplifier box (Costronics) and recorded using a Tektronics TDS 2012c oscilloscope (microsecond to millisecond timescale) and a National Instruments (NI USB-6211) DAQ card (millisecond to second timescale). The decays observed are the average between 500 and 1000 averages laser pulses. The data was processed using home-built software based on Labview.

Transient absorption experiments were measured while submerging the catalyst-loaded $TiO_2$ films into $N_2$-purged aqueous solutions (with or without 0.1 M TEOA buffered at pH 7).

### Definition of $t_{50\%}$

$t_{50\%}$ is defined as the time required for the signal amplitude (ΔO. D.) to decrease half of its initial value (full width half maximum). A detailed description of the calculation of $t_{50\%}$ for the direct electron transfer and the recombination reaction is provided in the figure captions of Figure S2 and S4, respectively.

### Density functional theory (DFT) calculations

The molecular structures were optimised in vacuum without any symmetry constrains, using the crystal structure provided as starting point geometry when available. The presence of local minimum was confirmed by the absence of imaginary frequencies. All calculations were carried out using the Gaussian 09 program[S5] with the Becke three parameter hybrid exchange, Lee Yang-Parr correlation functional (B3LYP). All atoms were described by the 6-311G(d) basis set. All structures were input and processed through the Avogadro software package.[S6]

### Calculation of the amount of catalyst loaded onto $TiO_2$

The number of catalyst molecules loaded per $TiO_2$ particle was estimated by UV-visible absorption spectroscopy (Perkin Elmer Lambda 35 UV/Vis spectrophotometre) after desorbing the catalyst from the nanoparticles by dipping a catalyst-loaded $TiO_2$ film (thickness: 4 μm, area: 1 $cm^2$) into a 0.1 M NaOH solution and comparing the absorption spectra with that of a solution containing a known amount of the cobalt catalysts.

The number of catalyst molecules attached onto a $TiO_2$ film was found to be 3.3 x $10^{16}$ for Co1, 3.9 x $10^{16}$ for Co2 and 4.0 x $10^{16}$ for Co3.

We have calculated the number of $TiO_2$ nanoparticles in a film, taking into account their volume.

Volume of 1 nanoparticle: $V_{part} = \frac{4}{3} \cdot \pi \cdot R^3 = \frac{4}{3} \cdot \pi \cdot (10x10^{-9})^3 = 4.2x10^{-24} m^3$, where $V_{part}$ corresponds to the volume of the nanoparticle ($m^3$) and R is the radius of the nanoparticle (m).

Volume of the film: $V_{film} = A_{film} \cdot L_{film} \cdot (1 - P_{film}) = 10^{-4} m^2 \cdot 4x10^{-6} m \cdot (1 - 0.6) = 1.6x10^{-10} m^3$, where $A_{film}$ is the area of the film ($m^2$), $L_{film}$ is the thickness of the film (m) and $P_{film}$ corresponds to the film porosity (60%)[S7].

The number of nanoparticles in a film was calculated as: $\#_{part} = \frac{Vfilm}{Vpart} = \frac{1.6x10^{-10}}{4.2x10^{-24}} = 3.8x10^{13}\ TiO_2\ nanoparticles/film$.

Thus, the number of cobalt molecules per $TiO_2$ particle was found to be approximately 900 for Co1, 1000 for Co2 and 1050 for Co3.

a)

**Co1**

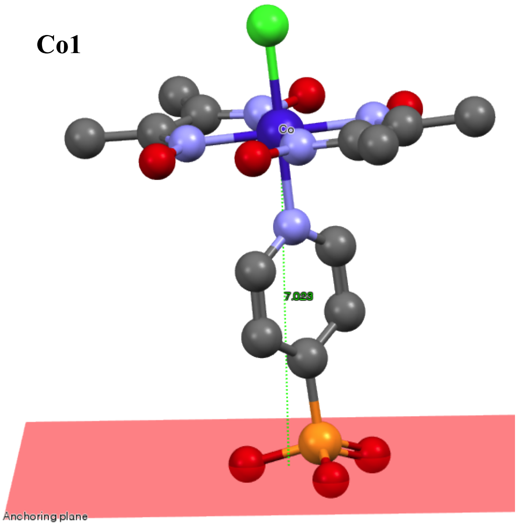


b)

**Co2**

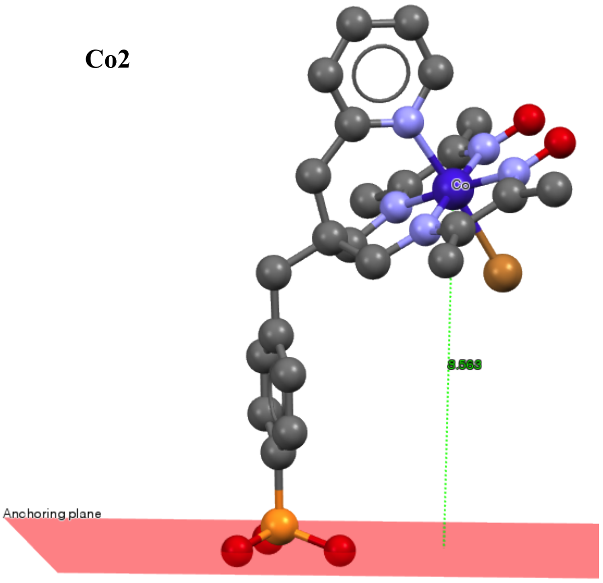


c)

**Co3**

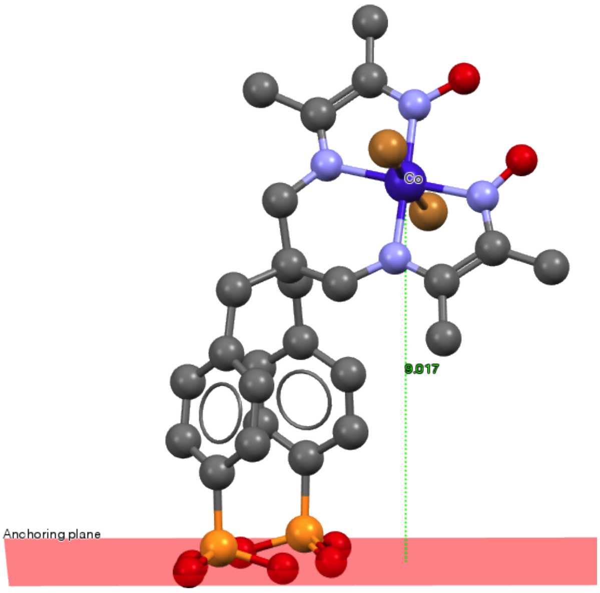


Figure S1. Graphical representation optimised geometries obtained by DFT means at B3LYP/6-311G(d) level of theory of (a) **Co1**, (b) **Co2** and (c) **Co3**. Distances were calculated from the cobalt metal centre to the anchoring plane formed by the three oxygen atoms in the phosphonic acid group using Mercury software package.

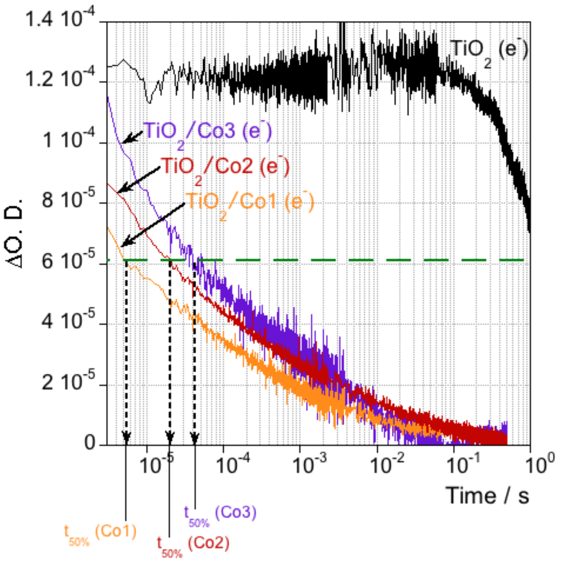


Figure S2. Transient absorption decays corresponding to photoexcited electrons in the $TiO_2$ for bare films (black trace), and when functionalised with **Co1** (orange trace), **Co2** (red trace) and **Co3** (purple trace), measured in the presence of TEOA (0.1 M, buffered at pH 7) as hole scavenger. **Calculation of $t_{50\%}$ for the direct electron transfer:** $t_{50\%}$ in $TiO_2$ films functionalised with the cobalt catalyst has been calculated by taking time values when ΔO. D. is half of that observed in $TiO_2$ films in the presence of TEOA as hole scavenger (green slashed line), by considering that the maximum electron density in $TiO_2$ is achieved when no catalyst is attached onto the surface. Note that light scatter limitations prevented data collection for time delays < 5 μs, half times were determined using the unfunctionalised $TiO_2$ as reference.

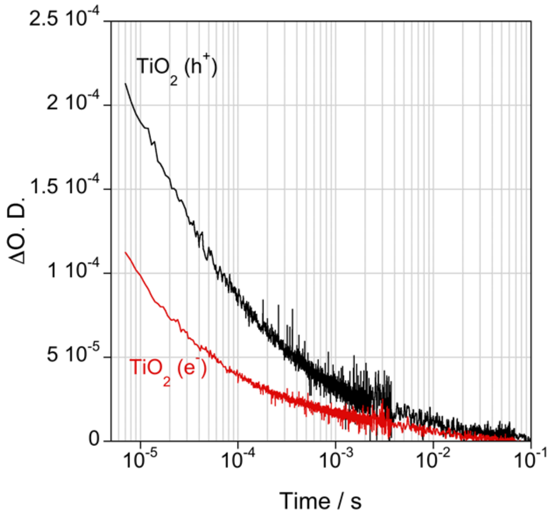


Figure S3. Transient absorption decays corresponding to photoexcited electrons (red trace) and holes (black trace) in a bare $TiO_2$ film, measured under $N_2$ purged water in the absence of hole scavenger. The excitation wavelength was 355 nm (350 μJ $cm^{-2}$, 1Hz) and the probing wavelengths were 900 nm for electrons and 460 nm for holes.

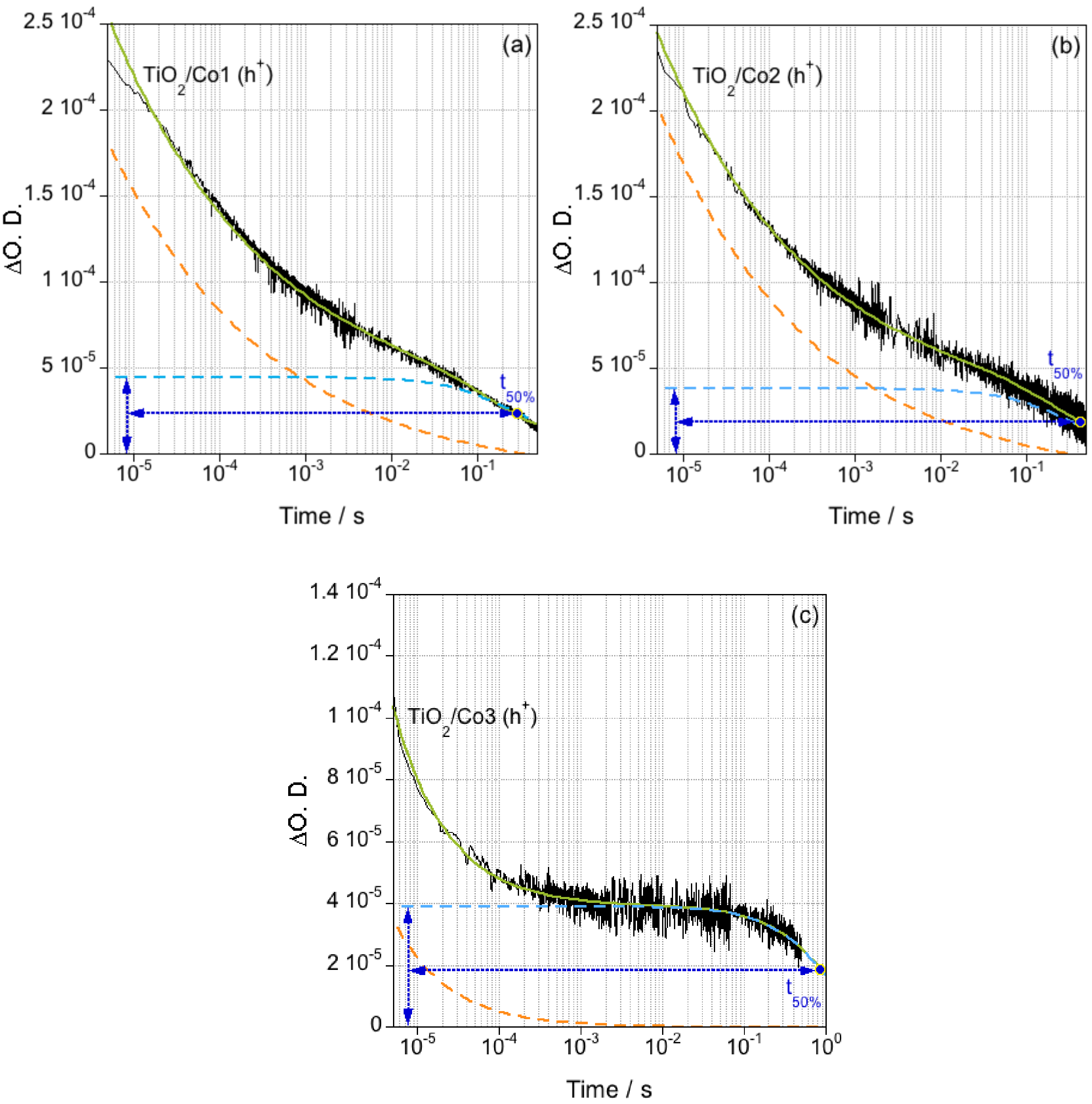


Figure S4. Transient absorption decays of $TiO_2$ functionalised with (a) **Co1**, (b) **Co2** and (c) **Co3**, measured in $N_2$ purged water. The red traces correspond to phototexcited electrons ($\lambda_{probe}$ = 900 nm), while the black traces are assigned to holes ($\lambda_{probe}$ = 460 nm). The samples were excited at 355 nm with a laser intensity of 500 µJ/cm$^2$. The decay of photoexcited holes was fitted by a combination of a power law equation (ΔO.D. α $t^{-\alpha}$, α(Co1) ≈ 0.23, α(Co2) ≈ 0.24, α(Co3) ≈ 0.6; orange slashed line) and a mono exponential equation (blue slashed trace). **Calculation of $t_{50\%}$ for the electron recombination:** $t_{50\%}$ of the recombination reaction was calculated by taking time values when ΔO. D. is half of that of the initial amplitude of the mono exponential fitting, assigned to the recombination of long-lived holes in the semiconductor with electrons transferred to the catalyst.

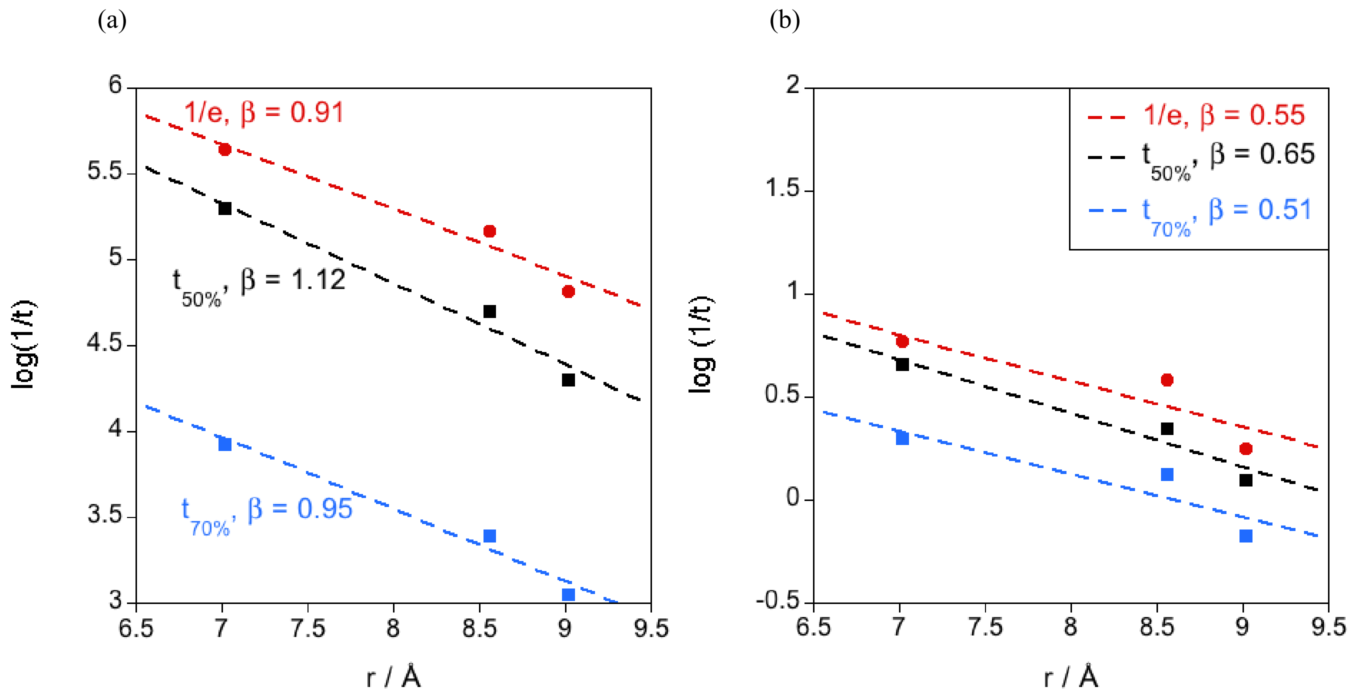


Figure S5. Comparison of the electron transfer kinetics when employing 1/e (red trace), $t_{50\%}$ (black trace) and $t_{70\%}$ (blue trace) of (a) the direct electron transfer, and (b) the recombination reaction between the singly reduced molecular catalyst and the $TiO_2$.

Table S1: Comparison of the electron transfer kinetics from $TiO_2$ to the molecular catalysts by using 1/e, $t_{50\%}$ and $t_{70\%}$.

| | Co1 | Co2 | Co3 | $\beta_{direct}$ |
|---|---|---|---|---|
| 1/e | 2.3 x $10^{-6}$ s | 6.8 x $10^{-6}$ s | 1.5 x $10^{-5}$ s | 0.91 |
| $t_{50\%}$ | 5.0 x $10^{-6}$ s | 2.1 x $10^{-5}$ s | 4.7x$10^{-5}$ s | 1.12 |
| $t_{70\%}$ | 1.2 x $10^{-4}$ s | 4.0 x $10^{-4}$ s | 8.9 x $10^{-4}$ s | 0.95 |

Table S2: Comparison of the kinetics of the recombination reaction between the singly reduced molecular catalysts and the holes accumulated at the valence band of $TiO_2$ by using 1/e, $t_{50\%}$ and $t_{70\%}$.

| | Co1 | Co2 | Co3 | $\beta_{recombination}$ |
|---|---|---|---|---|
| 1/e | 0.17 | 0.26 | 0.56 | 0.55 |
| $t_{50\%}$ | 0.22 | 0.45 | 0.8 | 0.65 |
| $t_{70\%}$ | 0.5 | 0.75 | 1.5 | 0.51 |

Table S3. Reduction potentials for the molecular catalysts and free Gibbs energy for the direct electron transfer from the semiconductor to the molecular catalyst, and the recombination reaction between the reduced catalyst and the semiconductor, represented in V vs NHE.

| | Co1 | Co2 | Co3 |
|---|---|---|---|
| $E_{p/2}$ ($Co^{III/II}$) / V vs NHE[a] | 0.00 | 0.04 | 0.22 |
| $E_{p/2}$ ($Co^{II/I}$) / V vs NHE[a] | –0.56 | –0.49 | –0.52 |
| $\Delta G^0$ (direct electron transfer) / eV[b] | 0.6 | 0.64 | 0.82 |
| $\Delta G^0$ (recombination) / eV[c] | 2.6 | 2.56 | 2.38 |

[a]Values were determined from cyclic voltammograms recorded in TEOA/$NaSO_4$ electrolyte (0.1 M each, pH 7) using a glassy carbon working electrode at 100 mV $s^{-1}$ and 25 °C. [b]Difference in energy between the conduction band of $TiO_2$ and the first reduction potential of the catalyst ($Co^{III}/Co^{II}$), assuming a $TiO_2$ conduction band at pH 7 of -0.6 V vs NHE.[S8, S9] [c]Difference in energy between the first reduction potential of the catalyst ($Co^{III}/Co^{II}$) and the valence band of the semiconductor ($TiO_2$ valence band being at 2.6 V vs NHE).

**Additional references**

[S1] F. Lakadamyali, E. Reisner, *Chem. Commun.* **2011**, *47*, 1695.
[S2] N. M. Muresan, J. Willkomm, D. Mersch, Y. Vaynzof, E. Reisner, *Angew. Chem. Int. Ed.* **2012**, *51*, 12749.
[S3] J. Willkomm, E. Reisner, *in preparation*.
[S4] S. Ito, T. N. Murakami, P. Comte, P. Liska, C. Grätzel, M. K. Nazeeruddin, M. Gratzel, *Thin Solid Films*, **2008**, *5156*, 4613.
[S5] M. J. Frisch, G. W. Trucks, H. B. Schlegel, G. E. Scuseria, M. A. Robb, J. R. Cheeseman, G. Scalmani, V. Barone, B. Mennucci, G. A. Petersson, H. Nakatsuji, M. Caricato, X. Li, H. P. Hratchian, A. F. Izmaylov, J. Bloino, G. Zheng, J. L. Sonnenberg, M. Hada, M. Ehara, K. Toyota, R. Fukuda, J. Hasegawa, M. Ishida, T. Nakajima, Y. Honda, O. Kitao, H. Nakai, T. Vreven, J. A. Montgomery, J. E. Peralta, F. Ogliaro, M. Bearpark, J. J. Heyd, E. Brothers, K. N. Kudin, V. N. Staroverov, R. Kobayashi, J. Normand, K. Raghavachari, A. Rendell, J. C. Burant, S. S. Iyengar, J. Tomasi, M. Cossi, N. Rega, J. M. Millam, M. Klene, J. E. Knox, J. B. Cross, V. Bakken, C. Adamo, J. Jaramillo, R. Gomperts, R. E. Stratmann, O. Yazyev, A. J. Austin, R. Cammi, C. Pomelli, J. W. Ochterski, R. L. Martin, K. Morokuma, V. G. Zakrzewski, G. A. Voth, P. Salvador, J. J. Dannenberg, S. Dapprich, A. D. Daniels, Farkas, J. B. Foresman, J. V. Ortiz, J. Cioslowski and D. J. Fox, *Gaussian 09, Revision B.01*; Gaussian Inc, Wallingford CT. **2009**.
[S6] M. D. Hanwell *et al. J. Cheminfor.* **2012**, *4*, 17.
[S7] M. Grätzel, *J. Photochem. Photobiol. C*, **2003**, 4, 145.
[S8] G. Rothenberger, D. Fitzmaurice, M. Gratzel, *J. Phys. Chem.* **1992**, 96, 5983.
[S9] B. Enright, G. Redmont, D. Fitzmaurice, *J. Phys. Chem.* **1994**, 98, 6195.
